# Novel autoantigens immunogenic in COPD patients

**DOI:** 10.1186/1465-9921-10-20

**Published:** 2009-03-12

**Authors:** Petra Leidinger, Andreas Keller, Sabrina Heisel, Nicole Ludwig, Stefanie Rheinheimer, Veronika Klein, Claudia Andres, Jürg Hamacher, Hanno Huwer, Bernhard Stephan, Ingo Stehle, Hans-Peter Lenhof, Eckart Meese

**Affiliations:** 1 Department of Human Genetics, Medical School, Saarland University, Building 60, 66421 Homburg/Saar, Germany; 2 Center for Bioinformatics, Saarland University, Building E.1.1, 66041 Saarbrücken, Germany; 3 Department of Pneumology, Inselspital, 3010 Bern, Switzerland; 4 Department of Cardiothoracic Surgery, Voelklingen Heart Center, 66333 Voelklingen/Saar, Germany; 5 Department of Clinical Haemostaseology and Transfusion Medicine, Medical School, Saarland University, Building 75, 66421 Homburg/Saar, Germany; 6 Department of Pneumology, Medical School, Saarland University, Building 91, 66421 Homburg/Saar, Germany

## Abstract

**Background:**

Chronic obstructive pulmonary disease (COPD) is a respiratory inflammatory condition with autoimmune features including IgG autoantibodies. In this study we analyze the complexity of the autoantibody response and reveal the nature of the antigens that are recognized by autoantibodies in COPD patients.

**Methods:**

An array of 1827 gridded immunogenic peptide clones was established and screened with 17 sera of COPD patients and 60 healthy controls. Protein arrays were evaluated both by visual inspection and a recently developed computer aided image analysis technique. By this computer aided image analysis technique we computed the intensity values for each peptide clone and each serum and calculated the area under the receiver operator characteristics curve (AUC) for each clone and the separation COPD sera versus control sera.

**Results:**

By visual evaluation we detected 381 peptide clones that reacted with autoantibodies of COPD patients including 17 clones that reacted with more than 60% of the COPD sera and seven clones that reacted with more than 90% of the COPD sera. The comparison of COPD sera and controls by the automated image analysis system identified 212 peptide clones with informative AUC values. By *in silico *sequence analysis we found an enrichment of sequence motives previously associated with immunogenicity.

**Conclusion:**

The identification of a rather complex humoral immune response in COPD patients supports the idea of COPD as a disease with strong autoimmune features. The identification of novel immunogenic antigens is a first step towards a better understanding of the autoimmune component of COPD.

## Background

Chronic obstructive pulmonary disease (COPD) is a common pulmonary affection, which is characterized by a range of pathophysiological changes including airflow limitation based on an obstructive bronchiolitis and persistent inflammation with neutrophils, macrophages, B and T lymphocytes and dendritic cells, a mucociliary dysfunction, apoptosis, and on structural changes in the airways causing emphysema, and by extrapulmonary systemic effects [1-3]. The overall global prevalence of COPD in adults that are 40 years or older is approximately 10% [4]. While COPD is currently the fourth leading cause of death worldwide it is expected to be the third leading cause of death in 2020 [5]. Besides, COPD is a major factor for disease-related loss of quality of life, health expenditure and loss of productivity.

Various host and environmental risk factors are supposed to contribute to the development of COPD. Host factors include airway hyperresponsiveness and aberrant lung growth. Several candidate genes are found to contribute to the individual risk. Environmental risk factors include smoking as the first cause followed by air pollution and occupational dust or chemicals [6].

COPD shares many clinical and pathophysiological features with autoimmune diseases [7]. There is strong evidence for an active adaptive T-cell response in COPD patients [8]. Antielastin antibody and T-helper type 1 responses characterize emphysema as an autoimmune disease [9]. The most recent evidence for a prevalence of IgG autoantibodies in COPD patients further supports the idea of a strong autoimmune component in COPD [10]. However, there is rather limited information on the nature of antigens reacting with autoantibodies in COPD patients. In this study we identified a large number of different antigens immunogenic in COPD patients and determined how many antigens reacted with each COPD serum. Likewise, we determined how many COPD sera reacted with each antigen. Additionally, we compared the reactivity of each antigen between COPD sera and control sera of healthy individuals. The identification of novel immunogenic antigens that allow differentiation of COPD patients from controls is a first step towards the development of new diagnostics and therapies as recently suggested [10].

## Methods

### Patient's sera

Blood samples were obtained from the Departments of Pneumology and Hemostaseology of the Saarland University with patients' informed consent. Serum was isolated from the blood samples and stored as aliquots at -70°C. The patient sera stem from 17 patients with COPD. Spirometrical data and data on smoking behavior are summarized in Table 1. Control sera were obtained from 60 healthy blood donors. None of the included blood donors suffered from pre-existing diseases according to their medical records and as determined by medical examination prior to blood drawing. Information on gender and age of all blood donors is given in Table 2.

**Table 1:** Information on COPD patients

**patient**	**diagnose**	**sex**	**age**	**GOLD**	**FeV1 %**	**BMI**	**smoking behaviour**
1	COPD, Emphysema	m	63	GOLD IV	26,5	18,6	former smoker, 100 py
2	COPD	f	72	GOLD II	75,7	27,5	former smoker, py unknown
3	COPD, Emphysema	f	77	GOLD IV	33,7	18,6	former smoker, 30 py
4	COPD	f	54	COPD IV	tracheotomy	23,4	former smoker, py unknown
5	COPD	f	55	GOLD III	45,6	21,4	40 py
6	COPD, Emphysema	m	67	GOLD IV	28,7	29,3	former smoker, py unknown
7	COPD, Emphysema	f	71	GOLD IV	30,3	27,4	former smoker, 50 py
8	COPD, Emphysema	f	55	GOLD IV	17,3	22,9	former smoker, py unknown
9	COPD	f	71	GOLD II	64,7	34,3	former smoker, 100 py
10	COPD	f	56	GOLD IV	15,3	20,7	former smoker, 20 py
11	COPD	f	57	GOLD IV	21,2	21,7	former smoker, 20–25 py
12	COPD	m	80	GOLD III	39,9	24,3	120 py
13	COPD	f	69	GOLD IV	21,3	30,4	former smoker, 120 py
14	COPD	m	55	GOLD IV	28,7	21,2	former smoker, 30 py
15	COPD	f	73	GOLD II	55,6	42	former smoker, 20 py
16	COPD	m	59	GOLD IV	27,5	21	former smoker, 30 py
17	COPD, Emphysema	f	52	GOLD IV	20,1	12,5	former smoker, 30 py

*Definition of abbreviations*: COPD = chronic obstructive pulmonary disease; GOLD = Global Initiative for Chronic Obstructive Lung Disease; m = male; f = female; FeV1 = forced expiratory volume in one second; BMI = Body Mass Index; py = pack years

**Table 2:** Gender and age of patients and controls

	**COPD sera**	**normal sera**
**number**	17	60
**male (%)**	29.4	68.3
**female (%)**	70.6	31.7
**age, mean**	63.9	38.7

### Protein macroarray screening

We assembled 1827 different immunogenic clones from high-density protein macroarrays consisting of 38,016 *E. coli *expressed proteins from the hex1 library [11] by carrying out a pre-screening with sera of patients with different human diseases. These 1827 *E. coli *expressed proteins were screened in duplicates with 17 COPD sera and 60 normal control sera. In brief, macroarrays were washed twice with TBSTT (TBS, 0.05% Tween 20, 0.5% Triton X-100) and 4 times with TBS. After blocking with 3% non-fat dry milk powder in TBST (TBS, 0.05% Tween 20), macroarrays were incubated over night with sera 1:1000 diluted in blocking solution (3% non-fat dry milk powder in TBST). After the incubation, sera were stored for the second incubation round. Three washing steps with TBST were followed by incubation with stripping solution at 70°C. Macroarrays were washed twice with TBST and 4 times with TBS. Incubation with blocking solution was followed by a second round of serum incubation over night. Macroarrays were washed three times with TBST, and incubated with secondary antibody (rabbit anti-human IgG, IgA, IgM-Cy5 (H+L)) 1:1000 diluted in blocking solution. Macroarrays were washed four times with TBST, twice with TBS and scanned by the GE Healthcare Typhoon 9410 scanner. The evaluation of the scanned protein arrays was carried out by the image analyzing software tool AIDA version 4.15 and by a newly developed computer-aided analysis procedure.

### Computational analysis of seroreactivity patterns

**Figure 1:**
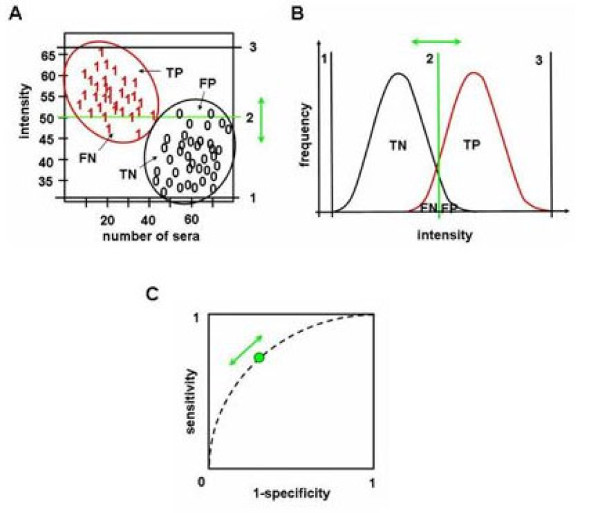
**The separation of intensity values of COPD and control sera is exemplarily shown for an arbitrary antigen *A***. A: Intensity values of each single COPD (1) and control (0) serum for antigen *A *are exemplarily shown. The position of the threshold (vertical green bar (2)) determines the number of true positives (TP), true negatives (TN), false positives (FP) and false negatives (FN). The vertical bars 1 and 3 indicate the minimal and maximal thresholds. B: The two curves represent density estimations of intensity values of COPD patients (red curve) and controls (black curve) for antigen *A *corresponding to Figure 1A. C: The specificity (TN/(TN+FP)) and sensitivity (TP/(TP+FN)) of a test are visualized by the receiver operator characteristics (ROC) curve. The performance of the test can be represented by the area under the ROC curve (AUC). Here, the threshold is represented by the green circle. The values for sensitivity and specificity can be modified by moving the threshold.

Since manual inspection offers only a subjective and binary analysis of reacting clones, we developed an automated image analysis procedure. After hybridizing the arrays with the different sera, our approach computes the intensity value for each clone on the arrays. Since all clones were spotted in duplicates, the mean value of the two replicates was assigned to each clone. The evaluated antibody profiles were normalized using quantile normalization to minimize between-array-effects. To access the "value" of an antigen with respect to its ability to separate COPD sera from control sera, we calculated the area under the Receiver Operator Characteristics curve (AUC) for each antigen *A *as follows: the normalized intensities of all control and COPD sera were used as threshold values. For all thresholds *t*, we considered COPD sera with intensity value above *t *as true positives (TP), COPD sera with intensity value below *t *as false negatives (FN), control sera with intensity value below *t *as true negatives (TN), and control sera with intensity value above *t *as false positives (FP). Likewise for all thresholds, specificity (TN/(TN+FP)) and sensitivity (TP/(TP+FN)) were computed. Please note that in some cases the classification has to be inverted. In these cases, diseased sera with intensity value below *t *are considered as 'true positives' (TP). The Receiver Operator Characteristics (ROC) curve shows the specificity as function of one minus the sensitivity. AUC values can range from 0 to 1. An AUC of 0.5 for a spot means that the distribution of intensity values of COPD sera and control sera can not be distinguished. The more the AUC value of an antigen differs from 0.5, the better this antigen is suited to separate between the two serum groups COPD and control. AUCs of 1 or 0 correspond to a perfect separation of spots generated by COPD and control sera. Antigens with AUC values > 0.5 show higher intensity values in COPD sera than in control sera. Antigens with AUC values < 0.5 show higher intensity values in control sera than in COPD sera. A graphical representation of the AUC value computation is provided in Figure 1.

## Results

### Visual analysis of protein macroarrays

**Figure 2:**
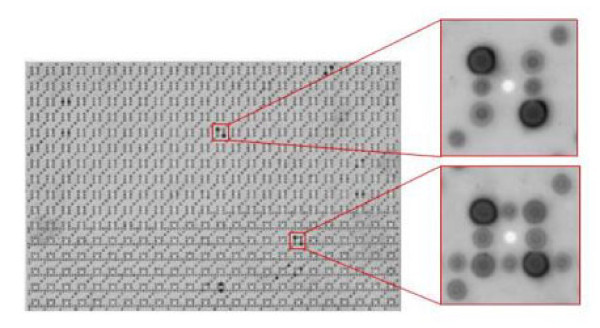
**Example for a protein macroarray analyzed with serum**. Since all clones are spotted in duplicate, any antigen – antibody reactivity is indicated by two signals.

Out of a fetal brain cDNA expression library encompassing more than 38,000 different peptide clones, we compiled a set of 1827 immunogenic clones by screening serum pools of various human diseases. To identify antigens reactive with COPD sera, these peptide clones were screened with 17 sera of patients with COPD and a control group of 60 sera of healthy blood donors. An example of a protein macroarray analyzed with serum is shown in Figure 2. The reactivity of each peptide clone was analyzed by visual evaluation utilizing the image analyzing software AIDA. Each COPD serum detected on average 63 peptide clones. The six most reactive sera identified more than 70 peptide clones per serum and the three least reactive sera identified less than 40 peptide clones per serum. In total, all 17 sera together detected 381 different peptide clones that reacted with autoantibodies of COPD patients.

Next, we asked how frequent does any of the peptide clones react with sera of COPD patients. We detected 17 clones that reacted with more than 60% of all tested COPD sera and seven clones that reacted with more than 90% of all tested COPD sera.

### Computational comparison between COPD sera and controls

**Figure 3:**
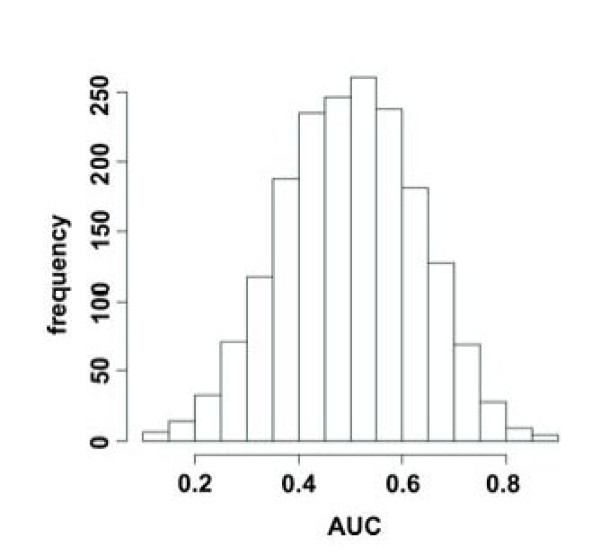
**Frequency of antigens according to their AUC value**. AUC values of all antigens were calculated for the classification COPD sera versus control sera. The distribution demonstrates a large number of antigens with AUCs < 0.3 and > 0.7.

Autoantibodies are not only found in diseased persons but are also common in healthy individuals. Therefore we analyzed the seroreactivity of antigens not only for COPD patients, but also for healthy blood donors as controls. To perform a low biased evaluation of the arrayed antigens, we utilized our newly developed computer-aided image analysis procedure. This automated evaluation approach allows to compare the seroreactivity of COPD and controls for each antigen. We computed the area under the Receiver Operator Characteristics curve (AUC) by comparing seroreactivity (intensity values) of COPD and control sera for each clone. The overall distribution of AUC values for all 1827 clones is shown in Figure 3. In the following we only examined clones with AUC values < 0.3 or > 0.7 because these clones are best suited to separate COPD from control sera. We found 212 peptide clones, including 67 clones that represent in frame sequences and 145 peptide clones that represent out of frame sequences. The 67 in frame clones represent 58 different genes. Comparison of the autoantibody profiles between male and female controls did not show a statistically significant influence of gender. Likewise, we did not find a significant influence of the age on the autoantibody profiles of normal controls (data not shown).

**Figure 4:**
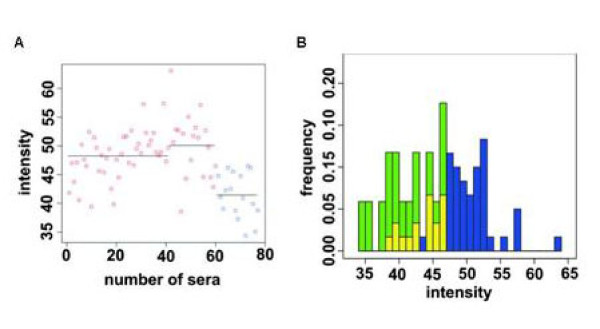
**Intensity values of FAM36A shown for all COPD and control sera**. A: FAM36A intensity values shown for each serum. Blue circles indicate sera of COPD patients and red circles indicate control sera. B: Distribution of seroreactivity signals according to their intensities. Seroreactivity signals that stem from controls are indicated in blue, and seroreactivity signals that stem from COPD sera are indicated in green. The overlaps are indicated in yellow. For this clone, signals with low intensities are mostly found with COPD sera and signals with higher intensities are mostly found with control sera.

**Figure 5:**
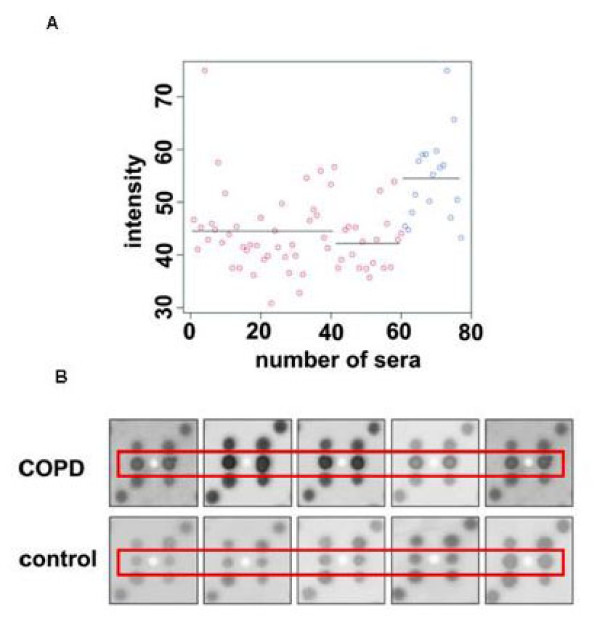
**Intensity values of MCM3 shown for all COPD and control sera**. A: MCM3 intensity values shown for each serum. Sera of COPD patients are indicated by blue circles and control sera are indicated by red circles. For this clone, signals with low intensities are mostly found with control sera and signals with higher intensities are mostly found with COPD sera. B: Example of seroreactivity signals. The position of the analyzed clone is indicated by red rectangles. Each clone of the array is spotted in duplicate. By visual analysis, this clone was found positive with all shown COPD sera and negative with all shown normal sera.

The best clone (AUC = 0.10) showed sequence homology to FAM36A but represents an out of frame sequence. The spot intensities of clone FAM36A for all sera and the distribution (frequency) of seroreactivity signals according to the spot intensities are shown in Figure 4. The best in frame clone (AUC = 0.87) showed sequence homology to MCM3 that has been identified as immunogenic antigen in colon and prostate cancer. The above mentioned clone FAM36A showed higher immunogenicity in control sera than in COPD sera. Clone MCM3, the best in frame clone, showed higher immunogenicity in COPD sera than in control sera (see Figure 5). In total, 40 of the 67 in frame clones showed higher immunogenicity in COPD sera than in control sera, whereas only 27 of the 67 in frame clones showed higher immunogenicity in control sera than in COPD sera. In addition, 17 clones showed homology to proteins that were identified as immunogenic antigens in various human cancers . These proteins included NME2, CDC42BPB, RPS2, PTBP1, SON, MCM3, CD320, VIM, CENPB, PDE4DIP, CCNL2, HMG-14, HSPD1, MAZ, RPL6, STUB1, and MBTPS1. Antigens YBX1, HMG-14, and CENPB which showed also informative AUC values are involved in the autoimmune disease systemic sclerosis. CENPB was additionally associated with the autoimmune diseases lupus erythematosus and rheumatoid arthritis [12-15]. Clones with homology to TRAF4 and NME2 have previously been associated with non-cancer lung diseases. More information on all 67 clones is summarized in Additional file 1.

### *In silico *analysis of the in frame peptide clones

Sequence motives like coiled coil, ELR, RGD, and granzyme B cleavage sites can be characteristic for autoantigens [16-18]. We analyzed the 58 different genes with the statistical gene analysis tool "GeneTrail" [19]. Our analysis revealed that 20.7% of the 58 sequences are containing coiled coil motives, whereas only 11.3% of all human gene sequences show such motives. We found approximately 10% more sequences with ELR motives in our test set, i.e. the 58 genes, as compared to the training set. Predicted granzyme B cleavage sites were found in 43.1% of the sequences in our test set and in 40.4% of the sequences in the reference set. In contrast, RGD motives were found in only 5.2% of the sequences in our test set but in 7.5% of the sequences in the reference set.

Out of the 58 genes, eight genes namely RPL37, RPL35, RPS27A, RPL23A, RPL6, RPS2, RPS6, and LOC388720are involved in the KEGG (Kyoto Encyclopaedia of Genes and Genomes) ribosome pathway. RPS6 is also involved in the insulin signalling and the mTOR signalling pathway. MCM3 and VIM are involved in the pathways cell cycle and cell communication, respectively. GTF2B, also known as TFIIB, is a member of the pathway basal transcription factors. HSPD1 is involved in the two pathways Prion disease and Type I diabetes mellitus.

## Discussion

The concept of COPD as an autoimmune disease is supported by the recent findings of frequent IgG autoreactive antibodies in COPD patients [7-10]. In our study, we identified 381 antigens that are immunogenic in COPD patients by screening protein macroarrays with patients' sera followed by a visual evaluation. We identified seven clones that were reactive in more than 90% of all COPD sera. To overcome a bias possibly caused by visual evaluation of the protein macroarrays, we additionally evaluated the macroarrays with a computer aided image analysis procedure that ensures a standardized evaluation of the arrays. Here, we identified 212 peptide clones with informative AUC values < 0.3 and > 0.7 by comparing seroreactivities of COPD patients and healthy controls. Clones with informative AUC values offer themselves as future biomakers for COPD. The identification of biomarkers is however not the focus of our study. Biomarker evaluation requires a study design that tests antigens with informative AUC values for their reactivity against a large number of patients' sera and control sera in a prospective manner. The identified peptide clones provide the basis for such a prospective study. The clones with AUC < 0.3 and > 0.7 included 67 in frame clones, representing 58 different genes and 145 out of frame clones that are termed mimotopes. Mimotopes are defined as peptides capable of binding to the antibody but unrelated in sequence to the natural protein that the antibody recognizes [20]. Since some of the mimotopes yielded rather informative AUC values as for example clone FAM36A, it will be worthwhile to reveal the nature of the natural protein that react *in vivo *with autoantibodies of COPD patients.

As indicated above a larger number of clones identified in this study were previously associated with an immune response in cancer or with autoimmune diseases. In addition, the two genes TRAF4 and NME2 have been associated with non-cancer lung diseases. TRAF4 deficiency leads to tracheal malformation resulting in airflow limitations in TRAF4-deficient mice [21]. NME2 negatively regulates Rho activity through interactions with other proteins involved in the Rho pathway [22]. In embryonic mouse lungs, the inhibition of the Rho pathway leads *in vitro *to reduced lung bud formation after 48 hours [23]. Since none of the clones identified in our study has previously been associated with the development or progression of COPD, the newly identified antigens may give leads to as of yet not analyzed cellular processes underlying COPD. Several of the identified antigens are currently discussed as biomarkers including HSP1, MAZ, and RPS2 that are up-regulated in colorectal cancer, acute myeloid leukaemia, and astrocytoma, respectively [24-26]. In addition, future biomarkers may be identified among the antigens NME2, CDC42BPB, RPS2, PTBP1, SON, MCM3, CD320, VIM, CENPB, PDE4DIP, CCNL2, HMG-14, HSPD1, MAZ, RPL6, STUB1, and MBTPS1 all of which have been associated with different human cancers. However, none of the identified antigens has been introduced into clinical practice.

It remains to elucidate what mechanisms contribute to the immunogenicity of each antigen in COPD patients. One hypothesis is that the humoral immune response against disease associated antigens results from overexpression. Although there is some evidence for an association of overexpression and immunogenicity of antigens [27,28], conclusive experimental proof for a causative role of overexpression has still to be provided. Posttranslational modifications including altered protein folding and processing may also cause a humoral immune response against disease-associated antigens. In addition, mutations are discussed as cause for the humoral immune response against immunogenic antigens [29]. However, we analyzed the reactivity of COPD sera using a cDNA library expressed in *E. coli* that lack the post-translational modification machinery. In addition, the sequences are highly unlikely to contain specific COPD mutations. Several sequence motives including coiled coil, RGD, ELR and granzyme B cleavage sites have previously been associated with the immunogenicity of antigens [16-18]. We found a slightly elevated percentage of coiled coil motives, ELR motives, and granzyme B cleavage sites in COPD associated antigens. Single analysis of each of these antigens will be required to elucidate the contribution of these motives and other factors to the immunogenicity of the antigens.

## Conclusion

Our study provides clear evidence that COPD shows strong autoimmune features. In contrast to previous studies, we not only demonstrated the prevalence of IgG autoantibodies but determined the nature of antigens reacting with autoantibodies in COPD patients. The identification of novel immunogenic antigens that allow differentiation of COPD patients from healthy controls will help to contribute to improved diagnostics and therapies.

## Competing interests

The authors declare that they have no competing interests.

## Authors' contributions

PL and EM conceived the study. PL, SH, NL, SR, and VK carried out the macroarray screening. AK and CA did all computational and statistical analysis of the protein macroarrays. PL, AK, HPL, and EM wrote the manuscript. HH, BS, JH, and IS supplied all tested sera and revised this manuscript critically. All authors read and approved the final manuscript.

## Supplementary Material

Additional file 1 **Antigens in frame with AUC values < 0.3 and > 0.7. **This table summarizes information on all in frame clones with AUC values lower than 0.3 and higher than 0.7. Click here for file
